# A Quandary of Cuprum - Wilson’s Disease Disguising as Progressive Myoclonic Epilepsy

**DOI:** 10.7759/cureus.951

**Published:** 2017-01-01

**Authors:** Monika Sachan, Suman Kushwaha, Shah faisal ahmad Tarfarosh, Vineet Banga, Ashutosh Gupta

**Affiliations:** 1 Senior Resident, Department of Neurology, Institute of Human Behaviour and Allied Sciences (IHBAS), Delhi, India; 2 Associate Professor & HOD, Department of Neurology, Institute of Human Behaviour and Allied Sciences (IHBAS), Delhi, India; 3 Resident, Department of Neurology, Institute of Human Behaviour and Allied Sciences (IHBAS), Delhi, India; 4 Post DM Senior Resident, Department of Neurology, Institute of Human Behaviour and Allied Sciences (IHBAS), Delhi, India

**Keywords:** wilson's disease, myoclonic jerks, progressive myoclonic epilepsy, cognitive decline, cognition, cuprum

## Abstract

Although metals are indispensable for the production of articles in our daily usage, the deposition of these metals in human tissue is known to cause disease. However, it is not always the ingestion of abnormal amounts of lead, iron, or copper that makes our tissues morbid; our hereditary and metabolic issues are to be blamed as well. Wilson's disease is one such hereditary disease that creates chaos in tissues, usually the brain and liver, via deposition of abnormal amounts of copper in them.

While Wilson's disease almost seems to bring a picture of a young patient with dystonia and other extrapyramidal symptoms in our imagination, seizures are very uncommon in this disorder. Non-stimulus-sensitive myoclonic jerks along with cognitive decline as the initial presentation of this disease have never been reported until now. In fact, such a presentation would make the neurologist believe that the patient has some type of progressive myoclonic epilepsy (PME), thus, creating a dilemma. We report two such dilemmatic cases of Wilson's disease that disguised as PME.

## Introduction

Copper deposition in organs, like the brain and liver, is an anomaly, and it is a pathognomonic feature of Wilson's disease--a hereditary disorder with an autosomal recessive inheritance [1]. The central nervous system involvement in this disease usually presents with either extrapyramidal, cerebellar, or even cerebral features [2]. Notably, it has been observed that only 6% of the cases of this disease present with seizures. Moreover, it is very unlikely that the seizures are of myoclonic variety, and if a patient of Wilson's disease presents with myoclonic jerks, these can be cortical or subcortical [3]. Undoubtedly, there is no such case report of Wilson's disease in the medical literature with pure myoclonic jerks as the initial presenting feature. We present two such cases (from different families) of this disease who had myoclonic jerks followed by a cognitive decline as the primary presenting clinical feature, thus, disguising as progressive myoclonic epilepsy (PME).

## Case presentation

Our first patient is a boy of 18 years who presented with a year-long history of myoclonic jerks that were generalized. These jerky movements were not sensitive to any stimulus. According to the parents of the patient, he had no abnormal developmental milestones. The scholastic performance of the patient had been poor for the previous ten months. This was succeeded by difficulty in speech as well as tremulousness of both arms for the past three months. The patient's past history was negative for dysphagia, dystonia, ataxia, hematemesis, jaundice, melena, any blood transfusion, or alcohol/drug abuse. This patient has eight siblings; the first three male children in his family had expired as a result of jaundice in their early years of childhood. Furthermore, the younger sister of this patient was already a diagnosed case of Wilson's disease and was also having extrapyramidal symptoms, myoclonic jerks, and cognitive decline.

**Figure 1:**
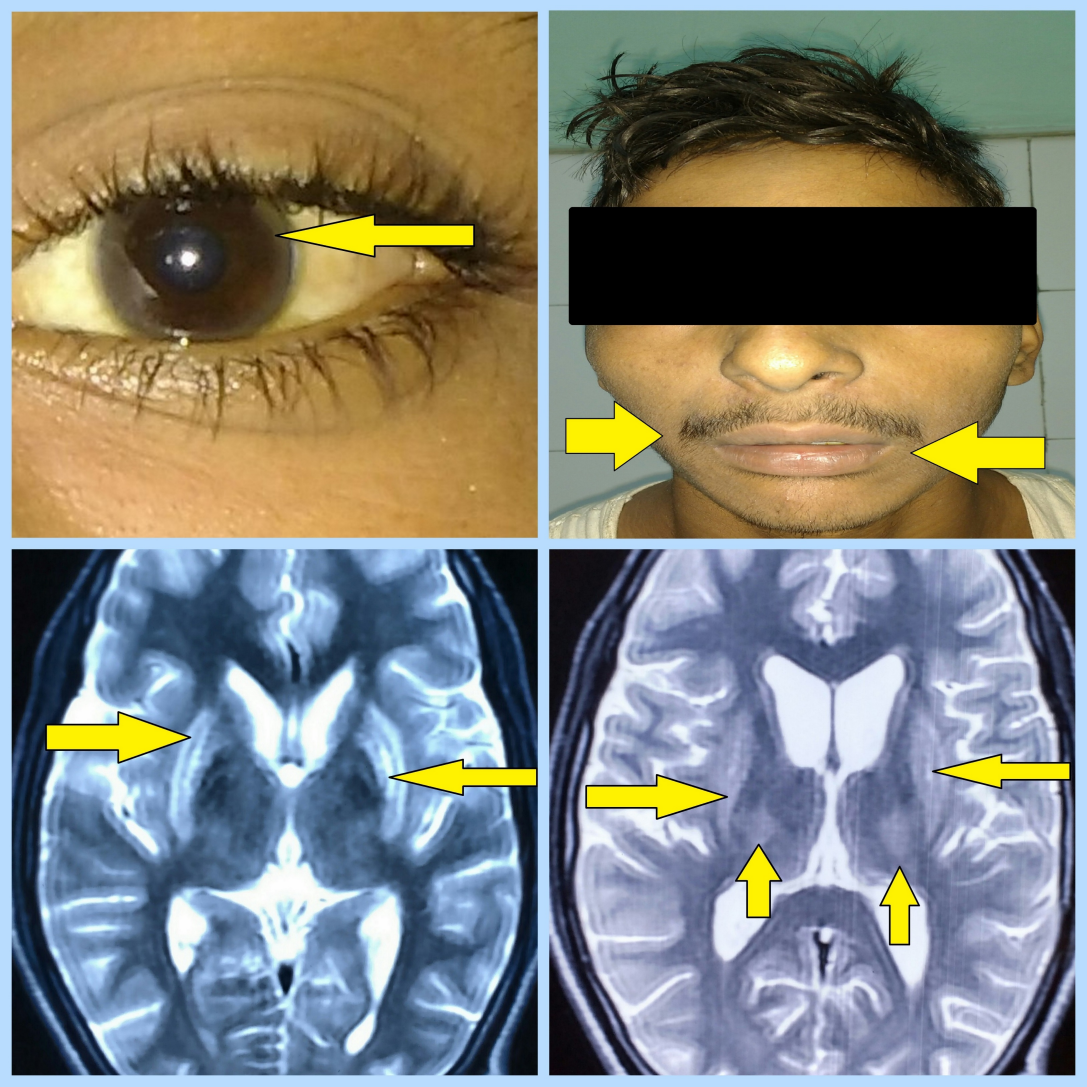
Figure Showing Kayser-Fleischer Rings, Mask-Like Facies, and Hyperintensity of Basal Ganglia and Surrounding Brain Gray Matter on MRI

On examination, it was found that the patient had bilateral action tremors along with spasticity, brisk reflexes in the upper and lower limbs, and dysarthria. The routine laboratory investigations, i.e., complete blood counts, liver function tests, and kidney function tests were normal. However, the serum copper level was 180 µgm/dl, and the serum ceruloplasmin level was found to be 10 µgm/dl. In addition, the 24-hour urine copper was 100 µgm/dl. The ultrasonography of the abdomen demonstrated coarse hepatic echotexture along with enlargement of the spleen. Kayser-Fleischer rings were found on the slit-lamp examination of the patient's eyes. Magnetic resonance imaging (MRI) of the brain illustrated bilateral and symmetrical T2 and flair hyperintensity in the following brain areas: the lentiform nucleus, especially the globus pallidus, thalamus, as well as in the midbrain and in the pons (Figure 1).

**Figure 2:**
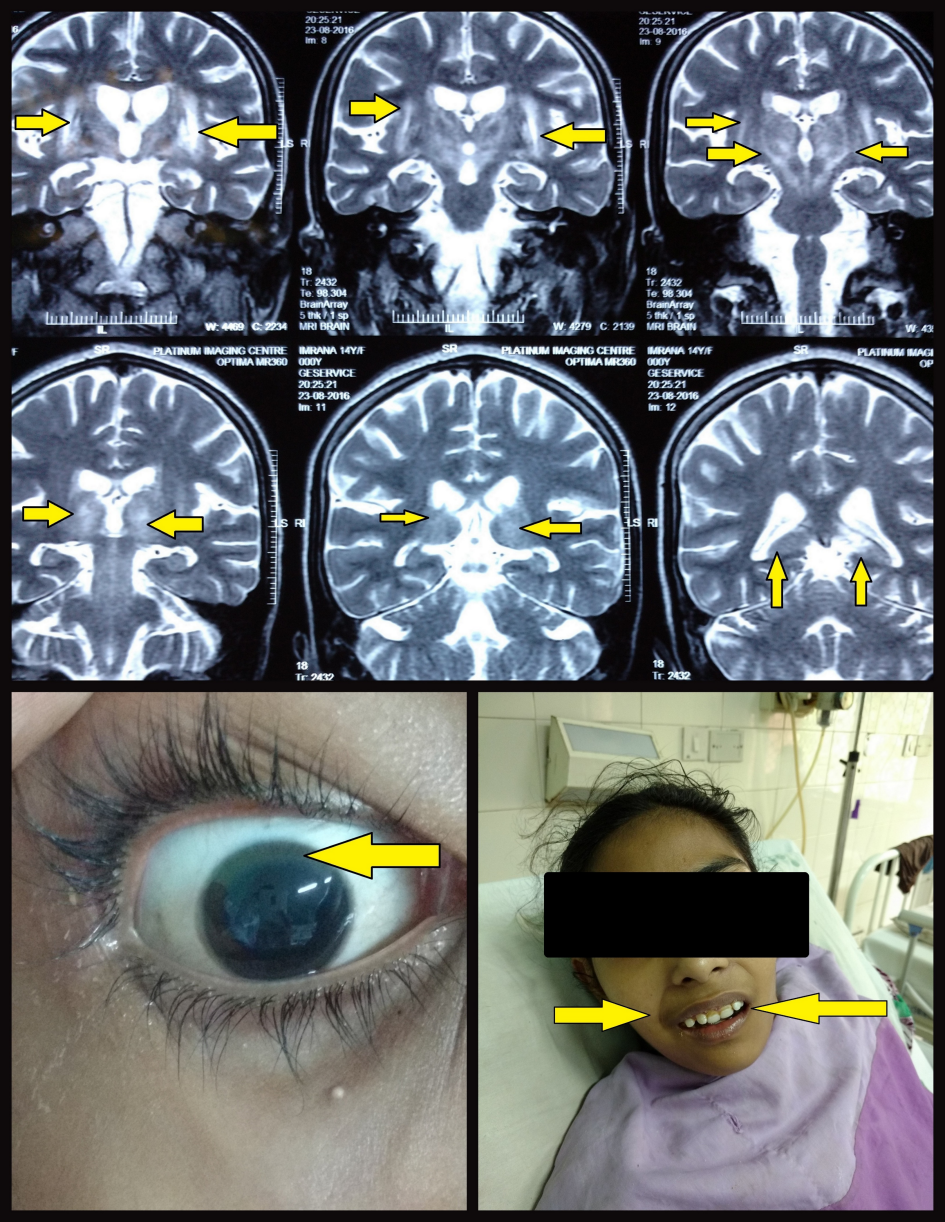
Figure Showing Hyperintensity of Basal Ganglia and Surrounding Brain Gray Matter on MRI, Kayser-Fleischer Rings, and Mask-Like Facies

The second case is a 14-year-old girl with absolutely normal developmental milestones. She presented with nearly the same complaints as described for the first case (Figure 2). Furthermore, she also had a positive family history of Wilson’s disease in two of her siblings.

Both of these two patients were managed conservatively after the diagnosis of Wilson's disease was made and showed significant improvement on the one-month follow-up. The patients agreed to participate and were explained the nature and objectives of this study, and informed consents were formally obtained. No references to the patients' identities were made at any stage during data analysis or in the report.

## Discussion

Almost half of the individuals suffering from Wilson’s disease have some kind of neurological dysfunction as their initial presenting feature [4]. Despite the fact that it is very less likely for such patients to have seizures, these have been reported to occur in some of the Wilson's disease patients. Nevertheless, most of these seizures are known to be of focal variety [3, 5]. Status epilepticus in such patients is rare; however, it can still occur [6].

Multiple mechanisms have been proposed for being responsible for the seizure activity in this disease. The first one is the inhibition of the membrane ATPase by direct deposition of copper in many parts of the brain, usually the basal ganglia. The other mechanism is suggested to be the brain tissue lesions due to copper deposition causing loss of neurons, laminar necrosis, gliosis, cavitation, and spongy degeneration of the cerebral cortex. This, in turn, results in focal seizure activity. Finally, the management of Wilson's disease by penicillamine leads to a deficiency of pyridoxine, which in itself is known to precipitate seizure activity. Notably, chelating therapy leads to the mobilization of copper. So, in Wilson's disease, although the seizures can take place at any stage, it mostly has been seen to start shortly when the chelating therapy is started [6]. However, in our case, the myoclonic jerky activity of the patients was present way before starting any treatment for the same.

There are only two case reports in the literature that have described generalized myoclonus in Wilson's disease. These patients had extensive lesions of white matter, and myoclonic jerks presented late and not early in the course of the disease [7-8]. In contrast, in our two cases, the 'myoclonic jerks, along with the decline of cognition' was the single most initial presenting combination of clinical features, thereby, disguising as PME and making a diagnostic dilemma for the neurologists. In one of these cases, the imaging illustrated extensive lesions of white matter in frontal, parietal, and temporal regions while preserving the fibers in the interhemispheric region of the cerebral cortex. In fact, necropsy of the patient showed significant alterations of the white matter, besides changes in parts of the basal ganglia and cortex [7]. No such white matter lesions were illustrated in the MRIs of our patients. In another such case reported by Mukherjee, et al. in 2016, some atypical symmetrical changes of white matter in the fronto-parieto-occipital area of the brain were seen. It was surprising to note that their case, although chronic, had diffusion restriction in the frontoparietal areas of both hemispheres [8]. No such features were observed in the imaging of our two cases. 

A case report, which was published very recently, described a Wilson's disease patient with a stimulus-sensitive myoclonus, a bilateral jerky tremor, and difficulty in walking along with a tandem gait [9]. On the contrary, our cases are extremely unique as both of them had generalized myoclonic jerks that were not at all sensitive to stimulus, had no tremors, and a normal gait.

The differential diagnoses of myoclonic jerks include the physiological, psychogenic, essential, secondary, and epileptic types of myoclonus [10]. Both of our patients were not healthy, and the jerky movements had no characteristic association--like jerks on falling asleep or with hiccups. So, we ruled out the physiologic myoclonus as it occurs in completely healthy individuals and is associated with hiccups, hypnagogic myoclonus, or even present as a physiological startle response [10].

Jerks in both of our cases were not distractible and were consistent over time. These had a gradual onset, had no day-to-day variability, and were not at all sensitive to stimulus. In contrast, the jerky movements as seen in psychogenic myoclonus are distractable, are inconsistent over time, have a sudden onset, have a day-to-day variability, and are usually sensitive to stimulus [10].

Jerks in essential myoclonus occur as an isolated finding and may be hereditary [10]; our patients had neither of these two features. We also ruled out the secondary causes of myclonus in our patients including any history of hypoxia, drugs, toxins, infections, focal nervous system damage, or any other hereditary neurological disease.

Finally, among the epileptic types of myoclonus, our cases resembled that of progressive myoclonic epilepsy syndromes. The characteristic features of these syndromes include myoclonic epilepsy, cognitive decline, progressive ataxia, and generalized tonic-clonic seizures (GTCS) [10]. Although our patients had the first two features, history or clinical examination did not depict any ataxia or GTCS. Furthermore, the myoclonus in PME is provoked by action or posture and is generally sensitive to noise, touch, or even light [10]. Our cases, on the other hand, had stimulus-insensitive myoclonus.

The new symptom combinations of Wilson's disease are being deciphered every now and then. We believe that in case a patient presents with any form of seizure-like activity along with cognitive decline and any single feature out of the of Wilson's disease symptoms, the copper and serum ceruloplasmin levels should be included in the routine investigations.

## Conclusions

Myoclonic jerks are rarely seen as the initial presenting manifestations of Wilson’s disease. So, while evaluating a patient having myoclonic jerks and cognitive decline and before labeling the case as PME, one should keep the diagnosis of Wilson's disease in mind. It should not be a chore to think of Wilson's disease as a differential only when a jaundiced young patient with acute dystonia arrives in the hospital emergency room. The atypical cases give us the insight to look beyond the patients' presenting complaint. In fact, we need to peer deeply into the history and clinical examination of the patients who may especially have a possible neurological, neurosurgical, or psychiatric disorder. The doctors who treat them need to be faster in the clinical suspicion and diagnosis than the head imaging they offer to their patients.
